# Nutritional Potentials of Atypical Feed Ingredients for Broiler Chickens and Pigs

**DOI:** 10.3390/ani11051196

**Published:** 2021-04-21

**Authors:** Olufemi Oluwaseun Babatunde, Chan Sol Park, Olayiwola Adeola

**Affiliations:** Department of Animal Sciences, Purdue University, West Lafayette, IN 47907, USA; bolufemi@purdue.edu (O.O.B.); park765@purdue.edu (C.S.P.)

**Keywords:** broiler chickens, diets, nutritional potentials, pigs, uncommon feed ingredients

## Abstract

**Simple Summary:**

Common feed ingredients such as corn, barley, wheat, soybean meal, and canola meal are used to feed broiler chickens and pigs in various countries around the world. However, due to rising costs and the need to practice sustainable animal husbandry, concerted efforts have been aimed at identifying and examining the nutritional potentials of atypical feed ingredients for pigs and chickens. Although there are some articles and reviews that discuss the potential of a single or few feed ingredients for either chickens or pigs, there has not been an extensive review that integrates information from several alternative feed ingredients for both species in one place. Therefore, this review aims to enumerate several feed ingredients that have shown prospects in supplying either one or more nutrients to pigs and chickens while reducing the dependence on commonly used feedstuff. In addition, feeding practices, merits, and limitations associated with these uncommon feed ingredients are discussed. Furthermore, practical applications of these alternative feed ingredients in relation to either pigs or chickens are briefly examined.

**Abstract:**

Diets play an important part in monogastric nutrition. This is because diets are comprised of various feed ingredients that supply energy and nutrients required by broiler chickens or pigs for normal growth and development. The main feed ingredients used for formulating diets for pigs and chickens are comprised of cereals and oilseed meals. Corn and soybean meal (SBM) are mostly used in North America for animal feeds. However, due to geographical locations, availability, and cost, ingredients such as wheat, barley, and canola meal are often used for feeding pigs and chickens. Overdependence on common ingredients such as corn and SBM for decades has resulted in rising costs of animal production. Therefore, the need has risen to examine the potentials of alternative feed ingredients capable of supplying the required energy and nutrients for monogastric animals. Research has been carried out to identify and evaluate several uncommon feed ingredients and their utilization by broiler chickens and pigs. Thus, this review enumerates the nutritional potentials of feed ingredients in 4 main nutritional classes using information from articles in peer-reviewed journals. Feeding practices, advantages, and limitations of using certain uncommon feed ingredients are discussed. In addition, species-specific factors in terms of practical applications are explored.

## 1. Introduction

Diets are one of the important considerations in the production of pigs and broiler chickens. Usually, a diet comprises various feed ingredients that supply energy and nutrients such as crude protein (CP), fats, fiber, minerals, or vitamins required for adequate growth by broiler chickens and pigs. Cereals and oilseed meals form the major portion of animal diets, as they supply energy, protein, and other nutrients. In the United States and much of North America, corn and soybean meal (SBM) are the major feed ingredients used in the formulation of animal diets. Other ingredients such as wheat, barley, canola meal, and distillers’ dried grains with solubles are used in Canada, Europe, South America, and other parts of the world [1].

However, there is competition for these feed ingredients in the food, biofuel, and bioprocessing industries, resulting in increased cost of over time [2,3]. Moreover, the need for agronomic sustainability has encouraged the planting of other crops such as triticale or sorghum with unique agronomic features including drought- or disease-resistance compared with corn or wheat [4]. Furthermore, the need for crop rotation with nitrogen-fixing plants such as pulses or legumes have increased the availability of alternatives to soybean [5]. In addition, co-products and by-products from the food or biofuel industries with little or no commercial value for humans have shown potential, to some extent, for use as feed ingredients for pigs and broiler chickens [1]. Indeed, feeding atypical ingredients to broiler chickens and pigs is a common practice in small-scale productions in Asia and Africa. Thus, depending on the geographical location, surrounding industries, or prevalent agronomic practices, certain feed ingredients that are not commonly used, but which have significant nutritional quality, could be added in diets for broiler chickens and pigs.

The use of atypical feed ingredients is not without challenges as several of these ingredients contain anti-nutritional factors (ANF) or toxins that hinder their use in animal feeding [2,6]. Moreover, inconsistency in the availability of some of these ingredients, federal regulations and approvals, and poor nutritional compositions in relation to common feed ingredients, limit their use on a commercial scale. However, extensive research has been conducted to identify and evaluate the nutritional prospects of uncommon feed ingredients for pigs and broiler chickens [7,8]. Several articles and reviews that report findings on the nutritional composition, nutrient utilization, and limitations associated with some of these ingredients for chickens or pigs have been published [2,9,10]. This information is however scattered across multiple articles or journals and may be species specific or relatively inaccessible to farmers who may require the information for adequate decision making.

Thus, the aim of this review was to combine and present information on the nutritional value of atypical feed ingredients for broiler chickens and pigs and in 4 main nutritional classes: protein-rich, energy-abundant, fiber-rich, and fat-delivering feed ingredients. The nutrient composition of these feed ingredients (Table 1) and the effects of including these feed ingredients in pig and broiler chicken diets on growth performance, and nutrient utilization are discussed. In addition, the advantages and limitations associated with each feed ingredient are outlined. Feeding practices such as inclusion levels in diets of pigs and poultry are discussed (Table 2), while species-specific factors and considerations in terms of applications are briefly explored.

## 2. Protein-Rich Feed Ingredients

Protein is necessary to supply amino acids (AA) for the building of muscle and for the repair of worn-out tissues. Sources of protein for diet formulation could be from plants or animal origins; however, plant-origin feed ingredients are commonly fed to pigs and broiler chickens due to relatively cheaper cost and availability. Animal-origin feed ingredients, such as fish meal or meat and bone meal, are generally expensive and are sourced from commodities which are in high demand as human food [2]. Soybean meal is the most widely fed protein source followed by canola meal; however, prices of SBM have increased due to competition for farming acreage with corn [2]. Alternatives to SBM or canola meal are available and are included in diets for broiler chickens or pigs at varying degrees. However, this is dependent on various factors such as presence of industries, prevalent crops in a region, or environmental factors. They can be classified under plant or animal-origin feed ingredients.

### 2.1. Plant-Origin Ingredients

Uncommon feed ingredients that are plant-based and rich in protein could be pulses such as faba beans (FB), field peas (FP), or chickpeas (CKP), which are mainly produced for consumption by humans; or by-products of the food-processing industries such as copra meal (CM) and palm kernel meal (PKM) [2].

#### 2.1.1. Faba Beans

**Figure 1:**
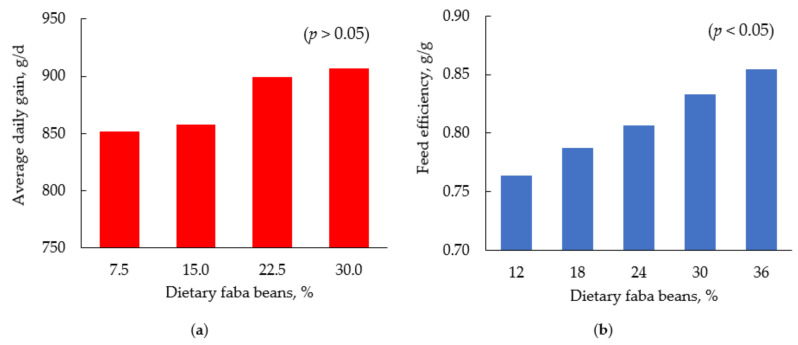
Growth performance of animals fed diets containing faba beans: (**a**) average daily gain (g/d) of growing pigs at 30 to 60 kg body weight [32]; (**b**) feed efficiency (g/g) of broiler chickens from 0 to 21 d post hatching [33].

Faba beans (*Vicia faba* L.) are leguminous crops grown in cool and temperate areas of Canada, Europe, and other parts of the world [9]. They have increasingly been favored as rotational crops due to their reduced environmental impact and nitrogen-fixing ability in the soil compared with other protein plants [5,9]. They are a rich source of protein (22–32% CP) [11,12]. Faba beans are rich in lysine but, as with other leguminous crops, are low in methionine [67]. This means that supplementation of crystalline methionine is required if FB are included in diets of broiler chickens and pigs [68]. The use of FB is on the increase due to the high cost of importing SBM in areas where it is not naturally grown. Furthermore, SBM is often obtained from genetically modified soybean cultivars, which is a concern for consumers and is not suitable for organic production thus, raising the need for alternatives in animal nutrition [69,70]. Faba beans are known to be high in ANF such as tannins, vicine, lectins, protease inhibitors, and convicine [6,31], which negatively impacts the growth performance and nutrient utilization of pigs and broiler chickens [6,71]. However, through efforts on breeding in Europe and other parts of the world, various FB cultivars with zero tannins concentration have been produced to further increase the potentials of its usage in the animal industry [9]. Inclusion of low-tannin FB in diets of grow-finish pigs did not impact growth performance negatively (Figure 1) [5,32], but improved digestibility of CP [11]. Similarly, including a combination of canola meal and FB in diets of grow-finish pigs supported performance and carcass quality comparable to SBM while improving meat characteristics [72]. The inclusion of FB varieties with low tannin and vicine in broiler diets improved the growth performance (Figure 1) [33] and energy and protein utilization of birds [6,73]. Kopmels et al. [13] reported improved performance, carcass properties, and yield when zero-tannin FB was included up to 15, 30, and 40% in the starter, grower, and finisher phases of broiler chickens, respectively. However, Tomaszewska et al. [70] reported a disturbance of the intestinal structure and tibia characteristics when raw low-tannin FB were fed to broiler chickens at the starter and grower phase. Pelleting of diets containing FB has been reported to improve the energy utilization of young chicks [71]. Furthermore, FB can be added at 20% and 30–40% to AA-balanced diets of broiler chickens and pigs, respectively, without negative impacts on their productivity [5,11,31,32,33].

#### 2.1.2. Field Peas

Field peas (*Pisum sativum*) are leguminous pulses that have been around for centuries and have mostly been consumed by humans. Field peas have a high nutritional quality that fall somewhere between corn and SBM and have the potential to substitute both of them in pigs and broiler chicken diets [34,74,75]. Field peas are high in lysine, but relatively low in methionine, tryptophan, and cysteine, and would require external supplementation of AA for use in monogastric animal diets [76]. They are grown in various parts of the world, but have been used to produce animal diets in Canada, Australia, and parts of Europe. Although the use of FP as pig or broiler chicken diets is not very common in the United States due to the availability of SBM and corn, they have regularly been fed to ruminants [35]. One major challenge with the use of FP is the variations in the nutritional profile among the available cultivars depending on where they are grown [14,77]. This has prevented the use of a uniform nutrient profile when formulating diets for pigs or chickens. Field peas also contain ANF such as tannins, trypsin inhibitors, lectins, saponins, and phytic acids at concentrations lower or similar to SBM [78]. Thermal treatment and extrusion of FP has been shown to deactivate most of the ANF present in the seeds [79,80]. The inclusion of FP in pig diets have been reported to improve growth performance [34] and digestibilities of starch, energy, and AA [79]. Similarly, Hugman et al. [81] reported that cold-pelleting and extrusion reduced the presence of trypsin inhibitors and improved the energy value and protein digestibility of FP in weanling pig diets. The inclusion of FP in diets of growing and finishing pigs has been shown to have no negative impact on carcass quality, composition, or pork palatability [35,82]. Field peas have not generally been included in broiler diets. However, Farell et al. [33] reported increased weight gain and feed efficiency in broiler chickens at 21 d fed FP up to 30%. McNeil et al. [78] also reported a slight increase in feed intake when FP was included in broiler diets at 10%, but a decrease in feed intake at 20%. The authors suggested that the presence of trypsin inhibitors might have played a role in the negative impact of high dietary concentration of FP on broiler chickens. Studies have suggested that if FP is properly processed, and adequate AA supplementation is carried out, it can be favorably included in diets for weanling pigs at 15–36% [34,36], growing and finishing pigs at 60–70% [35,37], and broiler chickens at 30% [33].

#### 2.1.3. Chickpeas

Chickpeas (*Cicer arietinum* L.) are leguminous plants commonly grown for human consumption. They have been available for centuries and are popularly grown and consumed in India, in the Mediterranean, and in Middle Eastern countries [15,83,84]. Proteins from CKP has also seen recent use in the production of plant-based meats and meat alternatives [85]. Feed grade CKP are available for animals and have been fed to animals as an alternative for SBM. Chickpeas are high in protein and energy as well as in minerals such as calcium and phosphorus, however, the nutrient composition varies depending on the cultivar [15]. The CP content of CKP ranges from 13–34%, however, they are limiting in methionine, cysteine, valine, threonine, and tryptophan [8,15]. As observed with other legumes such as FB and FP, CKP contains ANFs such as amylase and protease inhibitors, as well as lectins and polyphenols [15,86]. The use of extrusion and heat treatments on raw CKP has been reported to remove most of the ANF and increase its utilization in the diets of pigs and broiler chickens [38]. Inclusion of CKP in diets of weaned pigs at 30% reduced growth, feed efficiency, and protein digestibility, but did not impact feed intake and energy digestibility [16]. Similarly, inclusion of CKP in diets of finishing pigs did not hinder growth performance or protein digestibility [16,86], but improved fatty acid and carbohydrate digestibility while promoting a healthier ileal microbiota composition [87,88]. Replacement of SBM with CKP in diets of broiler chickens up to 12% did not affect performance and carcass qualities [39,89], but had negative impacts at 24% inclusion level [84]. Brenes et al. [90] reported improvements in protein and fat digestibility when broiler chickens were fed extruded CKP. Paszkiewicz et al. [69] reported a reduction in saturated fatty acids and an increase in the unsaturated fatty acids composition of the subcutaneous fat in broiler chickens when CKP was included in broiler chicken diets. It has been suggested that CKP can be included in diets of weaning pigs at 15% [16], finishing pigs at 30% [15,38], and broiler chickens at 12–15% [39,89].

#### 2.1.4. Copra Meal

Copra meal is a by-product of the coconut oil processing industry and is produced after the extraction of oil from dried coconut kernels [40]. Coconut palms, grown mostly in the tropical regions of Asia, Africa, and Central and South America, form a readily available source of energy and protein for local livestock industries [40]. Although CM may contain up to 15–25% CP, a bulk of which is arginine [91], its use in broiler or pig diets is limited due to low concentrations of some essential AA such as lysine and methionine [7]. Copra meal is also high in carbohydrates, although most of it is in the form of indigestible fiber and non-starch polysaccharides (NSP) such as cellulose, mannan, and galactomannan [91,92]. Due to high temperatures involved in the processing of CM, Maillard reactions occur, which damage lysine and other AA [40]. In order to improve the utilization and feeding value of CM for broiler chickens and pigs, interventions such as inclusion of enzymes, AA supplementation, and mechanical modifications have been suggested [91,93]. Inclusion of CM at 10–20% had negative impact on growth performance and nutrient utilization of broiler chicks at 21 d post hatching [41] due to the effects of high fiber and AA deficiency. Haetinger et al. [94] reported a reduction in energy digestibility with inclusion of CM in diets. A positive response on growth performance and intestinal characteristics was observed when a commercial diet diluted with CM and supplemented with a cocktail of carbohydrases and protease was fed to broiler chickens in the starter and finisher phases [95]. Feeding of mannanase-hydrolyzed CM improved performance and carcass characteristics of broiler chickens [96]. A negative impact on performance was observed with growing pigs fed above 30% CM [97]. The utilization of energy in CM by growing pigs has been favorably reported [51]. The partial replacement of SBM with CM in diets of finishing pigs did not affect carcass characteristics and composition of fatty acids in the backfat, but negatively affected nutrient digestibility [98]. Several studies have recommended that CM can be included up to 25% in diets of broiler chickens, 15% in weanling pigs, and 50% in grow-finish pigs, when lysine and methionine are supplemented [40,41,42]. Dietary supplementation with enzymes such as mannanase or phytase could also improve the utilization of fiber and phosphorus in broiler chickens and pigs fed CM [93,99,100,101].

#### 2.1.5. Palm Kernel Meal

Palm kernel meal is a by-product of palm oil processing after oil has been solvent extracted from the palm nut. Palm is mostly grown in the tropical regions of Asia, South America, and Africa, where PKM accounts for a huge part of diets formulated for broiler chickens and pigs [40]. The nutrient contents of PKM are usually dependent on the species of the palm and the method of oil extraction [102]. Palm kernel meal, with a CP content of 14–21%, is high in arginine and sulfur-containing AA, but low in lysine and tryptophan [18]. Palm kernel meal is also high in acid-detergent fiber (7–20%), mannans, and lignin, which results in a gritty and fibrous texture [92]. Most of the phosphorus present in PKM is bound to phytate, and therefore, the use of phytase is required to improve the utilization of minerals in pigs and broiler chickens [40,99,103]. More recently, PKM has been shown to improve the immunity and health of broiler chickens [104,105]. Feeding a combination of cassava root (CR) and PKM to finishing broiler chickens reduced feed cost and abdominal fat content, but negatively impacted feed intake and efficiency [106]. Inclusion of PKM in diets of pigs and broiler chickens may negatively impact growth performance and carcass qualities if exogenous AA such as lysine is not supplemented [107]. Growing and finishing pigs may be able to tolerate more than 20% of PKM, if the diet is balanced for standardized ileal digestible AA concentrations [40]. A combination of PKM and cassava peel meal improved growth performance of growing pigs as compared to feeding only cassava peel meal [108]. Jang et al. [109] observed that the growth performance and pork quality of grow-finish pigs were not affected when PKM was included in diets at 12% and supplemented with β-mannanase. Weanling pigs will also perform well with PKM inclusion levels up to 15% when diets are formulated with similar AA and energy concentrations [43]. Similarly, PKM can be included in broiler diets up to 40% without negative repercussions if properly combined with either lysine rich feed ingredients or crystalline AA supplementation [19,41].

### 2.2. Animal-Origin Ingredients

Some common protein-rich feed ingredients of animal-origin used in the diets of pigs and chickens include fish meal and meat and bone meal, which are expensive. Atypical animal-origin ingredients may be preferable as good sources of protein in animal diets, either due to their cheaper costs as by-products of meat processing plants, or lack of their consumption by humans. Some of these ingredients include poultry meal (PM), feather meal (FM), blood meal (BM), and insect meal (IM).

#### 2.2.1. Poultry Meal

**Figure 2:**
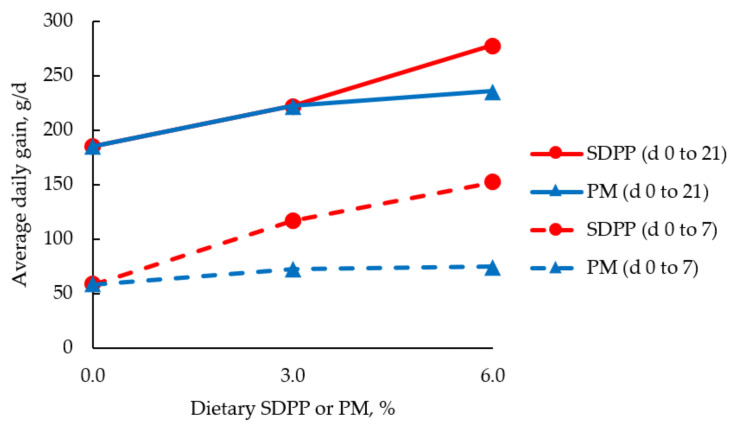
Average daily gain (g/d) of weanling pigs fed diets containing increasing concentration of spray-dried plasma protein (SDPP) or poultry meal (PM) [111]. The average daily gain of pigs fed SDPP (red circle and dashed line) was greater (*p* < 0.05) than those fed PM (blue triangle and dashed line) on 0 to 7 d; there was no difference in the average daily gain between pigs fed SDPP (red circle and solid line) and pigs fed PM (blue triangle and solid line) on 0 to 21 d.

Poultry meal is a by-product from poultry processing plants, which consists mostly of heads, feet, and inedible viscera of poultry after being rendered, dried, and ground [110]. With a CP of 52–60% [8], relatively cheaper cost, and good AA profile, PM has been touted as a potential substitute for fish meal in broiler and weanling pig diets. Poultry meal is also a good source of calcium and fat [111]. Several adjustments in the methods of processing PM and in the assurance of quality has given large improvements in the palatability and consistency of the feed ingredient [110], however, some variability still exists [112]. Currently, PM has been used favorably in the pet food industry as a protein source for dogs and cats [113]. Broiler chickens have also been successfully fed PM as a protein source with similar performance and nutrient utilization as birds fed fish meal or other protein sources [20,114,115]. Wisman et al. [44] reported that PM could replace up to 16% of the total CP in broiler starter diets without the need for external AA supplementation. Mahmood et al. [116] reported that the CP of broiler starter diets could be reduced up to 19% when supplemented with PM at 3% and protease at 200 g/ton of feed. Due to the need for highly digestible protein sources for weanling pig diets, PM has been evaluated as a potential ingredient in place of more expensive whey, fish meal, and plasma proteins [111]. Although weanling pigs fed diets containing PM throughout the nursery phase had similar growth performance with those fed plasma proteins, PM did not improve the feed intake and growth rates of pigs in the first week post-weaning (Figure 2) [111]. This observation indicated that there might be a limitation in using PM for pig diets immediately after weaning [110]. However, considering the entire nursery phase, the challenge was surmounted, and PM could be included in nursey diets by up to 10%. The utilization of energy in PM by weanling pigs was greater than corn and other protein sources [117]. Similarly, Vidyarathna and Jawaweera [118] suggested that PM could be included in diets of weaning and growing pigs at 10–26% without negative effects on growth performance. In diets of growing and finishing pigs, PM may not be valuable as the sole source of CP when compared with SBM due to the cost and a reported decrease in daily feed intake and weight gain of pigs [45]. However, the digestibility of AA in PM fed to growing pigs compares favorably with other animal protein sources [112].

#### 2.2.2. Feather Meal

Feather meal is another by-product of the poultry processing industry. Feathers account for about 5–7% of the live weight of mature birds and are composed of about 90% CP [119]. However, 88% of the protein present in feathers is in the inedible form of keratin, requiring processing to hydrolyze the keratinous bonds and make it usable for animal consumption [120]. After dressing of birds, feathers are collected and converted into FM either by steam hydrolyzation [121] or enzymatic processing [122]. Steam hydrolyzation has the disadvantage of damaging heat sensitive AA such as lysine, methionine, and tryptophan present in FM when it is carried out excessively and over long periods of time [123]. The availability of essential AA in FM is comparable with SBM, except for lysine [124]. However, differences in processing techniques and the composition of the raw feathers used may be responsible for the variability in the nutrient concentration of FM and in the utilization of AA and phosphorus by growing pigs [46]. Feather meal can be included up to 5–10% in diets for growing pigs without negative impacts on growth performance or carcass qualities [46]. However, supplementation of essential AA such as lysine, methionine, and other AA may be required [125,126]. Enzymatically processed FM has been reported to enhance growth performance of nursery pigs as comparable with plasma proteins when included up to 1.5% in nursery diets [47]. Similarly, FM may be added up to 6% in balanced diets of broiler chickens without a negative impact on productivity [44,48]. It should be noted that the use of drugs such as roxarsone in commercial poultry production could lead to the build-up of inorganic arsenic in poultry feathers which may be detrimental to both human and animal health when feathers are used as animal feed [127].

#### 2.2.3. Blood Meal

Blood meal is processed from blood collected after the slaughter of animals such as pigs, cattle, and poultry in processing plants. Blood meal is not commonly used in Europe and North America; however, it is a viable and cheap source of protein for local livestock industries in some countries in Asia and Africa [128]. Despite high concentration of CP (88–90%) [8,21], about 70% of BM is made up of red blood cell components which are poor in quality, while serum proteins make up the remaining 30% [129]. Blood meal is deficient in isoleucine, but is an excellent source of lysine, methionine, and cysteine [7,130]. Heat processing of BM damages a lot of the essential AA present, making them unavailable and limiting its use in animal diets [131]. Similarly, differences in the processing methods of BM have led to variabilities in the composition and digestibility of AA among products [132]. Squibb and Braham [131] reported decreases in growth performance of broiler chicks fed BM above 4% due to deficiency and imbalance of AA. With improvements in processing techniques, BM has garnered interest as a potential source of protein in combination with other CP sources in diets of pigs and broiler chickens. Ekwe et al. [133] observed that including bovine BM up to 15% in finisher diets for broiler chickens supported performance and nutrient utilization and was relatively cheaper than using fish meal as the sole source of protein. Donkoh et al. [49] observed improvements in growth performance of broiler chicks fed solar dried BM (7.5%) in combination with fish meal and without the inclusion of essential AA. Laboissiere et al. [134] observed that a combination of FM and BM could be included in diets of broiler chickens at 9% in the pre-starter and starter phase without negative effects on performance and nutrient digestibility. However, processing methods for BM could reduce the nutrient utilization in broiler chickens. Similarly, M’ncene et al. [128] reported no negative impact on growth performance and nitrogen utilization when pigs were fed BM in combination with molasses at 10% in a corn-soybean meal-based diet. Aderibigbe et al. [22] also suggested that BM could be used as an alternative protein source for growing pigs due to the high digestibility and utilization of nitrogen and AA. Spray-dried BM can be used in nursery diets for pigs to improve weight gain and feed efficiency [50].

#### 2.2.4. Insect Meal

The consumption of insects by humans has been in existence for centuries all over the world [135]. Insects are an ectothermic, rapid growing, and highly feed efficient species that can convert waste from both plants and animals into proteins and lipids [10]. Insect meal has been considered as the future of livestock production due to various factors including: (1) the increasing interest in sourcing for alternative protein source for animals, (2) the relatively expensive cost of existing protein sources such as fish meal, (3) the abundance of insects that could be used to supply proteins for animals, and 4) the ease at which IM can be produced from the industrialized mass-rearing of insects [135]. Insects are favorably compared to SBM or fish meal due to similar CP (40–60%) content [23]. However, defatting of insects is required to improve the CP content and palatability of IM [136]. Some common insects used in the production of IM include the black soldier fly, yellow mealworm, and the common housefly [136]. The complete or partial substitution of SBM with defatted IM (from black soldier fly larvae) in diets of piglets and growing pigs revealed no differences in growth performance, pork quality, sensory parameters, and an increase in nitrogen digestibility [137,138]. Growing pigs fed diets containing dried mealworm at 10% had increased or similar energy and AA utilization as compared to pigs fed fish meal, meat meal, or PM [139]. Similarly, Hwangbo et al. [24] included up to 20% IM (from housefly larvae) in diets of broiler chickens and reported improvements in carcass quality and growth performance of birds, while the replacement of fish meal with IM by 25% improved the protein efficiency ratio [140]. The inclusion of IM as a protein source for pigs and broiler chickens shows great potential and may eventually be accepted worldwide if legalities regarding safety in different regions are successfully developed. Currently, regulations in the European Union on feeding processed animal proteins hinder the use of insects as protein sources for animals. Moreover, there are concerns that insects may spread diseases to animals and humans because they are fed waste products [25]. Further articles that extensively review insect as an alternative protein source for pigs or chickens exist in peer reviewed journals [10,25,136].

## 3. Energy-Abundant Feed Ingredients

Feed ingredients that supply energy form the bulk of diets for pigs or broiler chickens and contribute substantially to the cost of diets for animal production. Corn is the most common energy-supplying feed ingredient in diets of pigs or chickens followed closely by wheat, barley, and sorghum. However, rising costs of corn and other cereals due to the competition with human consumption and arable land has led to the evaluation of other uncommon feed ingredients as a potential source of energy. Root crop meals such as CR and cereals like triticale have been extensively researched as energy sources. Similarly, by-products from the food industry such as bakery meal (BKM) and molasses have been considered as potential sources of energy for broiler chickens and pigs.

### 3.1. Cassava Root

Cassava is one of the most widely grown tuber and roots crop in the world. It thrives particularly well in the tropical regions of Africa, Asia, and South America, with Nigeria, Brazil, and Thailand being the world’s leading producers [141]. Cassava produces the most energy yield per unit of land area thus, making it one of the most energy dense crops available [142]. The cassava plant has the advantage of being resistant to drought, tolerant of poor soil conditions, and can maintain its nutritional qualities for a long time, even in the absence of adequate rainfall [143]. In some developing countries, cassava is regarded as a major carbohydrate supplying food, hence it is in high demand for human consumption. Cassava has been fed to animals in one form or another, with peels, leaves, or roots usually being fed to ruminants. Moreover, due to the high cost of importing corn in these developing countries, CR have been regarded as alternatives to corn in supplying energy to broiler chickens and pigs. It should be noted that CR is low in protein, some essential AA, vitamin A, iron, and zinc [143,144]. Additionally, cassava contains ANF such as cyanogenic glucosides that are poisonous to animals, which deters its use as an energy source in animal diets. Although there are sweet varieties with lower cyanide content, processing of the CR removes most of the cyanide present in the roots [145]. The metabolizable energy (ME) of CR has been reported to range from 3000 to 3300 kcal/kg in broiler chickens and from 2900 to 3400 kcal/kg in pigs [7,8,144,146]. Broilers fed CR were observed to have reduced feed intake and weight gain due to challenges with palatability, dustiness, bulkiness, gut-filling effect, and cyanide concentrations [142,147]. However, pelleting CR has been reported to improve the nutrient digestibility and growth performance of broiler chickens [147]. A range of 10–50% dietary CR, in combination with corn maintains the growth performance of broiler chickens [51,52]. Grow-finish pigs fed CR with CP intake up to 350 g /day showed acceptable growth performance and carcass qualities [53]. This indicates that with the proper supplementation of other nutrients, CR has the potential to serve as a major source of energy for pigs and broiler chickens.

### 3.2. Bakery Meal

Coproducts from the food industry has often been fed to pigs and chickens due to their potential as alternatives sources of nutrients. Bakery meal is obtained from the baking and food industry and is a combination of waste and unsalable portions from the processing of pasta, cakes, potato chips, breakfast cereals, cookies, bread, candy, inedible flours, or dried dough [148,149]. The different components are mixed, ground, and dried to produce BKM. However, there are inconsistencies in the chemical composition of nutrients and energy in BKM likely due to the vast range of commodities combined in the final product [148,150]. Bakery meal has an average sugar content of 70%, most of which are monosaccharides (glucose and fructose), and the disaccharide sucrose [148]. Bakery meal contains high concentrations of salt and may also contain high levels of NSP, if ingredients rich in fiber are in the final product. Bakery meal is a poor protein source, but its AA digestibility in growing pigs ranges from 70–80% and is reportedly similar to maize germ meal, hominy feed, and CR meal [151,152]. Due to the high content of energy derived from starch, sugars, and fat, BKM has been offered to weanling pigs and broiler chickens particularly in areas where large scale baking industries are available [148]. The ME of BKM for pigs approximately ranges from 3150 to 3850 kcal/kg in pigs [8,26,153] and from 3500 to 3900 kcal/kg for broiler chickens [7,151]. Although BKM is high in energy, its use as an energy source in commercial swine or broiler diets is limited due to the inconsistency in nutrient composition. Mackenzie et al. [54] suggested that BKM could be included in swine diets up to 10% without adverse effect on growth performance. Tiwari and Dhakal [154] replaced up to 75% of corn with BKM without adverse effects on growth performance in weaned pigs. Replacing corn-SBM diet for finishing pigs with a combination of BKM and restaurant food waste up to 50% had no negative impact on growth performance and carcass qualities except for meat color, which was paler than the control [155]. Similarly, including BKM up to 25% in broiler diets had no negative impact on growth performance, feed utilization, and litter condition [55]. From all indications, BKM has the potential to supply energy in animal diets; however, the challenge is the variability in the nutrient composition.

### 3.3. Triticale

**Figure 3:**
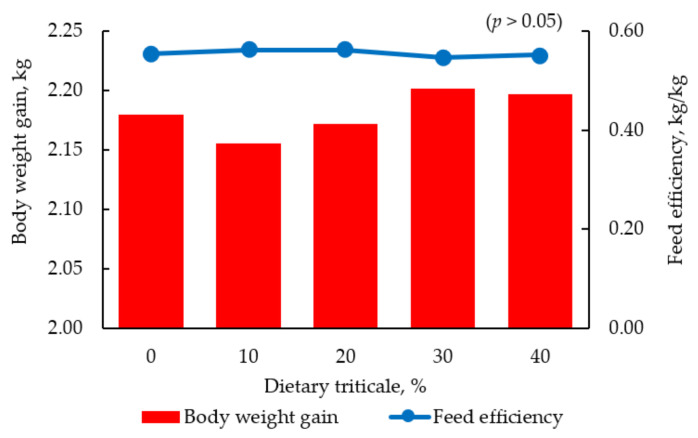
Body weight gain (kg) and feed efficiency (kg/kg) of broiler chickens fed diets containing increasing concentration of triticale from 1 to 42 d post hatching [57].

Triticale is a grain developed within the last century and is a cross between wheat and rye [56]. Triticale is more alike with wheat both in agronomic properties and nutritional value as compared with rye [4,156]. Triticale has been widely adapted to a wide range of environments with spring varieties grown mostly in parts of Africa, Asia, and South America while the winter varieties are grown in parts of Europe and North America [157]. In contrast to corn, triticale has a higher CP content and a better AA profile, making it a potential source of energy and CP in swine or broiler diets [156]. There is a lot of inconsistency in the nutrient composition of triticale due to the different cultivars available which limits the use of triticale in animal diets [17]. The presence of NSP, such as xylans and arabinoxylans that increase viscosity in the gut of pigs and broiler chickens, similarly limits its use in animal diets. However, supplementation with enzymes has been shown to alleviate this challenge to an extent [158]. The ME of triticale varies with different cultivars but ranges from 2900 to 3700 kcal /kg in broiler chickens and from 3200 to 3300 kcal/kg in swine [7,8,159]. When fed to pigs as the sole source of energy and protein, the ME ranged from 3080 to 3190 kcal/kg while the availability of isoleucine, methionine, and valine were comparable with corn [160,161]. The need for SBM was reduced by including triticale as the only grain in the diets of pigs thus, potentially minimizing feed cost [162]. This is due in part to the high CP and AA content of triticale as compared to other cereals hence reducing the quantity of SBM needed to meet the CP requirements of the pigs. There was no negative effect of feeding triticale on pork quality; however, the growth performance of pigs was negatively affected when fed above 40% [56]. Adeola et al. [163] reported reductions in energy utilization and dry matter digestibility, but not in protein digestibility and net protein utilization when corn was replaced with triticale from 0 to 100% in the diets of pigs. Supplementation of triticale with carbohydrases, crystalline AA, and the limit of its use below 50% may improve its use in swine diets. In broiler diets, a partial replacement of other energy sources with triticale may be the best way to improve its utilization with suggested recommendations at 40% (Figure 3) [57]. Supplementation of triticale with xylanase may increase its inclusion in broiler diets up to 60% without negative impacts on performance and nutrient digestibility [17].

### 3.4. Molasses

Molasses is a by-product from sugarcane processing and have been fed to animals for ages. Sugarcane has historically been grown in the southern region of the United States, and in parts of Brazil, Asia, Africa, and the Caribbean [164,165,166]. It thrives in temperate but mostly tropical regions of the world [167]. However, it has not been used recently in commercial diets for pigs or chickens due to more readily available feed ingredients and the reduced dependence on sugarcane for the processing of sugar in developed countries. Other challenges with molasses include its laxative effects on pigs and chickens and the difficulty with mixing diets when included above 20% [58]. Molasses are rich in sugars such as fructose, glucose, and sucrose, and have the advantage of increasing the palatability of diets due its sweetness. The inclusion of molasses in broiler diets up to 24% had no negative impact on their growth performance [59]. Bice et al. [168] suggested that cane molasses could replace cereal grains in broiler diets. In weanling pigs, molasses has been considered as an energy source in nursery diets in place of lactose due to its highly digestible sugar content. Mavromichalis et al. [60] observed that the total replacement of lactose with molasses had no negative impact on growth performance and nutrient digestibility in nursery pigs. In growing pigs, feed intake, growth performance, and carcass quality were reduced when molasses were fed above 400 g/d without adequate CP supplementation [169]. However, grow-finish pigs fed summer diets supplemented with cane molasses up to 11% recorded improved growth performance and reduced serum cortisol levels [58]. Finishing pigs fed a combination of molasses and royal palm nuts had improved weight gain and feed efficiency as compared to only molasses [170]. In developing countries where sugarcane is grown, molasses may play an important role in meeting some of the energy requirements of pigs or chickens if they are combined with rich protein sources.

## 4. Fiber-Rich Feed Ingredients

Fiber is an important part of animal nutrition and is responsible for increasing bulk of diets, promoting gut movement and health, and providing energy in the form of volatile fatty acids (VFA) to broiler chickens or pigs. Although challenges such as increased intestinal viscosity and water-holding capacity arise with feeding fiber the microbes present in the hindgut of broiler chickens and pigs are able to utilize fiber to some extent. Some common fiber-rich sources include wheat bran and soybean hulls. However, by-products from the processing of rice, oats, and sugar beets could serve as sources of fiber to livestock in areas where these crops are grown and processed. The use of rice bran (RB), sugar beet pulp (SBP), and oat hulls (OH) in ruminant nutrition is common. These have recently been introduced to diets of broiler chicken and pigs as a source of fiber with varying degrees of success.

### 4.1. Sugar Beet Pulp

**Figure 4:**
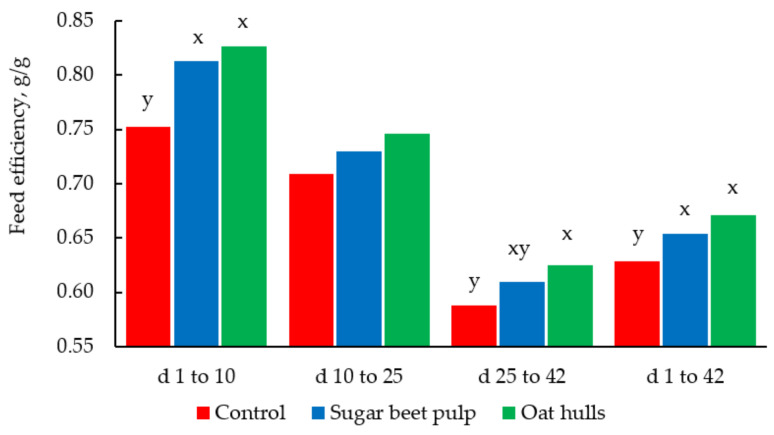
Feed efficiency (g/g) of broiler chickens fed diets containing sugar beet pulp or oat hulls from 1 to 42 d post hatching [62]. Means within the period without a common letter differ (*p* < 0.05).

Sugar beet pulp is a by-product of the sugar processing industry and is obtained after the juice in sugar beet has been extracted and the residue has been processed and dried [171]. Due to the high content of NSP including cellulose, hemicellulose, and pectin in SBP, it is in demand by the biofuel industries, leading to competition with the livestock industry [3]. Pigs can utilize the considerable amount of fermentable fiber in SBP as a source of energy because the low lignin content allows for easy fermentation in the hindgut [172]. The digestibility of the neutral and acid detergent fiber in SBP is high when fed to pigs [173]. When finishing pigs were fed SBP (inclusion rate of 15%) at the expense of barley, growth performance, and carcass characteristics were not impacted negatively and there were indications of improvement in gut health [173]. Supplementing SBP with a cocktail of carbohydrases such as xylanase, cellulase, and β-glucanase, improved the utilization of energy, protein, dry matter, and fiber as well as increasing the production of VFA in growing pigs [27]. Improvements in gut microbiota and gut health was also observed in growing pigs fed SBP [174]. A study by Gebbink et al. [175] introduced SBP to weanling pigs and there was some evidence that SBP supported the increased production of VFA and reduced the population of harmful bacteria in the gut; however, it did not affect growth performance of piglets. Sugar beet pulp may be included in diets of growing pigs up to 30% without deleterious effects if formulated with adequate dietary protein [61]. Fiber is usually considered an ANF in poultry due to the negative impact on performance and nutrient digestibility [176]. In a study where SBP was fed to broiler chickens for 42 d, feed intake, feed efficiency, and nutrient digestibility of birds were improved in the starter phase; however, feed intake was reduced in the finisher phase by 5.8% (Figure 4) [62]. This was attributed to the bulkier digesta induced by the pectin content of SBP, which consequently reduced passage rate and feed consumption in older birds. There are indications that fiber may be more beneficial to younger birds and that SBP may be considered as a fiber source in the formulation of starter diets for broiler chickens [62,177]. However, supplementing SBP (at 7.5%) with a cocktail of enzymes improves the digestibility of nutrients, development of the small intestine, and meat quality of broiler chickens at the finisher phase [178].

### 4.2. Rice Bran

Rice is one the most consumed crops in the world, and thus, there is an abundance of RB that is a by-product of the milling and processing of rice. Rice bran is high in fiber (20–25%), but also rich in proteins, lipids, vitamins, and minerals, and thus, it has been used in the livestock industry as animal feed [29]. However, due to the presence of lipids and lipases in RB, the occurrence of hydrolysis and oxidative rancidity is high. Similarly, RB contains ANF such as trypsin and pepsin inhibitors, and has most of its phosphorus bound as phytate, limiting its use in broiler chicken and pig diets [179]. The process of defatting and stabilization through heat treatment has reduced some of the challenges associated with RB, which results in increased shelf-life and potential as source of fiber and other nutrients [180]. Some studies have introduced RB in nursey pig diets and observed improvements in performance, dry matter digestibility, and gut health of weanling pigs similar with the effects of antibiotics [63,181]. It has been suggested that RB may possess some prebiotic properties that increase the proliferation of beneficial microbes in the gut of pigs and promote gut health [56]. Fan et al. [182] reported reduced inflammatory biomarkers and modulation of the intestinal barrier of finishing pigs fed increasing levels of RB as a replacement for corn. Although a reduction in growth performance and nutrient digestibility of broiler chicks has been observed with increasing RB levels and regardless of the supplementation of enzymes, it has been suggested that RB may be included 10–20% in broiler diets [64,183]. The negative impacts of RB in broiler chickens have been attributed to its fiber and phytate content and may continue to limit its usage in broiler diets. However, RB shows some potential to be utilized in swine diets as pigs are able to digest and utilize its nutrients to an extent.

### 4.3. Oat Hulls

Oat hulls are co-products from the processing of oats and are the outer and lighter shells of oat grains. They are very high in insoluble fiber and low in energy and protein [28,65]. In ruminant nutrition, OH are frequently used as roughage extenders when forages are in short supply. Similarly, OH has been included in diets of pigs and broiler chickens as a fiber source, carrier of micronutrients, and to reduce feed cost. However, because OH is low in energy, dietary fat is usually increased to meet the energy requirements of pigs and chickens [65]. Studies have shown that inclusion of OH to broiler diets improved feed consumption and starch digestibility, while weight gain and digestibility of other nutrients were not impacted [184]. The authors reasoned that feeding OH to broiler chickens, especially in the coarse form, increased the weight of the gut and its content, leading to an increased rate of passage and feed consumption [184,185]. Gonzalez-Alvarado et al. [62] also reported an increase in performance, nitrogen retention, and nutrient digestibility of broiler chickens fed OH regardless of the age of the birds (Figure 4). There was no detrimental effect on weight gain and AA digestibility, but energy utilization was reduced in broiler chickens when up to 30% wheat was replaced with OH [185]. In another study, inclusion of OH in corn-SBM-based diets of growing pigs had positive impacts on weight gain, feed intake, and VFA production, but negatively affected fat digestibility and cholesterol absorption [65]. Kim et al. [186] observed that supplementation of OH in nursery diets containing rice and animal protein reduced the occurrence of diarrhea in weanling pigs without a negative impact on growth performance. Similar observations were reported by Mateos et al. [66,187], and the authors suggested that including OH at 2% in nursery diets would not affect growth performance of piglets. The inclusion of OH in diets of pigs and broiler chickens could be beneficial considering some of the positive impacts observed in the animals and the added advantage of potentially reducing feed cost without impacting production.

## 5. Fat-Delivering Feed Ingredients

Fat is another important constituent of animal diet, as it supplies energy required by animals for growth and development. Depending on the geographical location, some form of plant or animal-based fat is included in animal diets. Soybean oil, lard, or tallow are some of the most commonly used fat-delivering feed ingredients [30]. Others include corn oil and choice white grease. Dietary fat is known to influence the quality of fat deposited in pigs, and thus, choosing appropriate dietary fat sources in diets for pigs is important [188]. An atypical fat-delivering ingredient that has been used in diets of pigs and broiler chickens is palm oil (PO).

### Palm Oil

Palm oil is the main product extracted from palm nuts and is rich in saturated fatty acids (FA) particularly palmitic acid. Palm oil is also rich in monounsaturated oleic acid, tocotrienol (part of the vitamin E subgroup), and carotenes, but is low in polyunsaturated FA [189]. Because of the high demand of PO for human consumption, it is not usually included in animal diets. Notwithstanding, with a gross energy of 9178 kcal/kg [190], PO has shown great potential in supplying the energy required by pigs and chickens. Inclusion of PO in swine diets improved the apparent digestibility of nitrogen, AA, and minerals in growing pigs [189,191], and the fat digestibility in weanling and growing pigs [30]. Similarly, PO had the highest production value in terms of economics and in reference to animal fat sources when fed to weanling and growing pigs [30]. Because the FA composition of dietary lipids directly influences the FA deposited in swine [190], PO has the advantage of maintaining hard fat in pork bellies due to its high concentration of saturated FA. Finishing pigs fed diets with PO had thicker backfat depth as compared with those fed soybean oil or tallow [192]. Palm oil is also used in broiler diets as a source of energy without negative impacts on performance [193,194]; however, carcass may contain high levels of saturated FA [195]. Feeding red PO to broiler chickens has been reported to reduce plasma cholesterol levels [196]. In Asia and Africa where palm trees are commonly grown, PO may be used as an energy supplement in diets of broiler chickens and pigs if it is cost effective.

## 6. Conclusions, Implication, and Future Research

The use of feed ingredients in animal feed will mostly be impacted by its nutrient composition, availability in a geographical location, and the economic cost of feeding them to animals. Most of the ingredients reviewed in this article have shown the potential to supply nutrients to pigs or chickens. However, their use may be limited by intricate characteristics of the animal species or the feed ingredients themselves. With the increasing human population and the consequent increase in the demand for food, every feed ingredient is a resource that could potentially help feed the population of the world. Therefore, research that evaluates and improves the utilization of these feed ingredients for monogastric nutrition should always be carried out. Lastly, it should be noted that the term ‘atypical’ is relative depending on the geographical location. Thus, feed ingredients selected by the authors for review are mostly atypical for diets of broiler chickens and pigs in the United States.

## Figures and Tables

**Table 1:** Nutrient composition of some atypical feed ingredients for broiler chickens and pigs.

Item	Gross Energy, kcal/kg	Crude Protein, %	Ether Extract, %	Crude Fiber, %	References
Faba beans	3800–4500	22–32	0.96–1.3	7.5–8.6	[8,11,12,13]
Field peas	4035–4500	20–31	0.9–1.3	6.2–12.7	[8,14]
Chickpeas	4200–4500	12–34	0.4–0.9	0.4–12.5	[15,16]
Copra meal	4199	21–22	3.0	12.8–16.4	[8,12,17]
Palm-kernel meal	4150–4350	14–21	3.8–8.5	17.9	[8,18,19]
Poultry meal	4080	50–60	15.9	- ^1^	[8,20]
Feather meal	5200–5500	79–89	6.8–9.1	0.32	[8,12,20]
Blood meal	5300–5473	88–92	0.8–1.5	-	[8,21,22]
Insect meal	4800	40–60	20–40	-	[23,24,25]
Cassava root	3450–3560	2.5–2.9	0.5–0.9	2.9–4.4	[8,12]
Bakery meal	4200–4558	12.3–13.6	8.1	-	[8,26]
Triticale	4316	13.6	1.8	2.5	[8]
Molasses	4223	4.8	0.2	-	[8]
Sugar beet pulp	3633–4050	8.1–9.8	0.8–1.0	17.3–19.9	[8,12,27,28]
Rice bran	3800–4100	14.4–17.3	3.1–3.5	20–25	[8,12,29]
Oat hulls	979	4.6–5.1	1.4	25.9–28.7	[7,28]
Palm oil	9100–9400	-	-	-	[8,30]

^1^ Nutrient composition values could not be found in literature for these feed ingredients.

**Table 2:** Recommended inclusion rate (%) in diets of pigs and broiler chickens.

Item	Broiler Chickens	Weanling Pigs	Grow-Finish Pigs	References
Faba beans	20–40	- ^1^	30–40	[5,11,31,32,33]
Field peas	15–36	60–70	30	[33,34,35,36,37]
Chickpeas	12–15%	15	30	[16,38,39]
Copra meal	25	15	50	[40,41,42]
Palm-kernel meal	30–40	15	10–20	[19,40,41,43]
Poultry meal	10–16	10	-	[44,45]
Feather meal	6	1.5	5–10	[44,46,47,48]
Blood meal	7.5	5–7.5	-	[49,50]
Insect meal	10–15	0–6% replacement of SBM ^2^	50–100% replacement of SBM	[10,24]
Cassava root	10–50% replacement of corn	-	50–100% replacement of corn	[51,52,53]
Bakery meal	25	-	10	[54,55]
Triticale	40–60	-	20–40	[17,56,57]
Molasses	24	100% replacement of lactose	11	[58,59,60]
Sugar beet pulp	3	-	30	[61,62]
Rice bran	10–20	10	-	[63,64]
Oat hulls	3	2	5–10	[62,65,66]
Palm oil	-	5	10	[30]

^1^ Recommended inclusion rate values could not be found in literature for these feed ingredients. ^2^ SBM = soybean meal.
